# Barriers and facilitators to implementing a regional anesthesia service in a low-income country: a qualitative study

**DOI:** 10.11604/pamj.2019.32.152.17246

**Published:** 2019-04-01

**Authors:** Matthew Ho, Patricia Livingston, M Dylan Bould, Jean Damascène Nyandwi, Françoise Nizeyimana, Jean Bonaventure Uwineza, Robin Urquart

**Affiliations:** 1 Department of Anaesthetics, Royal Prince Alfred Hospital, Sydney, Australia; 2 Department of Anesthesia, Pain Management and Perioperative Medicine, Dalhousie University, Halifax, Canada; 3 Department of Anesthesiology, University of Ottawa, Ontario, Canada; 4 Department of Anesthesiology and Intensive Care Medicine, University of Rwanda, Kigali, Rwanda; 5 Department of Surgery, Dalhousie University, Halifax, Canada

**Keywords:** Regional anesthesia, global health, knowledge translation, consolidated framework for implementation research (CFIR)

## Abstract

**Introduction:**

Regional anesthesia is a safe alternative to general anesthesia. Despite benefits for perioperative morbidity and mortality, this technique is underutilized in low-resource settings. In response to an identified need, a regional anesthesia service was established at the University Teaching Hospital of Kigali (CHUK), Rwanda. This qualitative study investigates the factors influencing implementation of this service in a low-resource tertiary-level teaching hospital.

**Methods:**

Following service establishment, we recruited 18 local staff at CHUK for in-depth interviews informed by the “Consolidated Framework for Implementation Research” (CFIR). Data were coded using an inductive approach to discover emergent themes.

**Results:**

Four themes emerged during data analysis. *Patient experience and outcomes:* where equipment failure is frequent and medications unavailable, regional anesthesia offered clear advantages including avoidance of airway intervention, improved analgesia and recovery and cost-effective care. Professional *satisfaction:* morale among healthcare providers suffers when outcomes are poor. Participants were motivated to learn techniques that they believe improve patient care. *Human and material shortages:* clinical services are challenged by high workload and human resource shortages. Advocacy is required to solve procurement issues for regional anesthesia equipment. *Local engagement for sustainability:* participants emphasized the need for a locally run, sustainable service. This requires broad engagement through education of staff and long-term strategic planning to expand regional anesthesia in Rwanda.

**Conclusion:**

While the establishment of regional anesthesia in Rwanda is challenged by human and resource shortages, collaboration with local stakeholders in an academic institution is pivotal to sustainability.

## Introduction

Despite advances in health care provision in low-HDI countries (defined by Human Development Index (HDI<0.8), perioperative mortality is at least threefold greater than in high-HDI countries (HDI > 0.8) [1]. This problem is particularly acute in sub-Saharan Africa, where the avoidable anesthesia mortality rate has been recorded as 1:504 in Malawi [2] and 1:133 in Togo [3]. Rwanda, a small, land-locked country in East Africa, is among the most densely populated nations in the world. Health care measures are poor, with annual health expenditure at $125 per capita (compared with the USA's $9,403 per capita) [4]. Regional anesthesia is an integral component of modern perioperative care. Potential safety benefits are particularly relevant in Rwanda where adverse outcomes from general anesthesia can occur due to oxygen and electricity failure, complications from failed airway management, equipment malfunction, and lack of pulse oximetry [3]. Studies demonstrate an association of regional anesthesia with improved analgesia, increased patient satisfaction, reduced costs, increased operating room efficiency and reduced post-anesthesia care unit (PACU) length of stay for specific surgeries [5]. While there is a paucity of data in low-HDI countries [6], it is reasonable to hypothesize these advantages are transferable, and possibly amplified in low-resource settings where additional benefits include: reduced use of anesthetic drugs in limited supply, cost reduction, and the ability to use simple, easily transportable equipment [7].

Despite this, regional anesthesia is underused in low-resource settings [8]. A survey of 147 Nigerian anesthesia providers [9] showed that while 92.9% regularly used spinal anesthesia, only 2.9% regularly used peripheral nerve block techniques. In this group, 47.1% of anesthesia providers have never performed a nerve block. Only 10-15% of patients in Rwanda receive regional anesthesia for lower limb surgery [10]. Prior to the establishment of a regional anesthesia service in 2016, peripheral nerve blocks were not regularly taught or practiced in Rwanda. The factors responsible for underuse of regional anesthesia in low-resource settings include lack of: equipment, drugs, designated space, training of anesthesia staff, skilled nursing support, knowledgeable administrative personnel, facility and logistics planning, patient and surgeon education, and quality assurance programs [6]. In response to an identified need, a regional anesthesia service was established at a tertiary hospital in Rwanda (see below). This study aims to understand the factors influencing establishment of the regional anesthesia service. The Consolidated Framework for Implementation Research (CFIR) was selected as a guiding framework for exploring implementation and addressing the research goal [11].

## Methods

Ethical approval was obtained from the Dalhousie University Research Ethics Board (2015-3642), and the University of Rwanda's (UR) Institutional Review Board (CMHS/IRB/078/2016).

**Setting:** anesthesia is an emerging specialty in Rwanda. In the aftermath of the 1994 genocide, Rwanda had one anesthesiologist for 8 million people. In 2006, the Canadian Anesthesiologists' Society International Education Foundation (CASIEF) and the American Society of Anesthesiologists Global Humanitarian Outreach (ASAGHO) partnered with UR to support anesthesia teaching for postgraduate physicians [12]. This partnership continues today as both CASIEF and ASAGHO send regular volunteer anesthesiologists to teach in the 4-year Masters of Anesthesia curriculum. This program has been growing successfully to 31 anesthesia resident trainees, as of January 2018.

**Establishment of regional anesthesia service:** in early 2016, Canadian and Rwandan study investigators partnered to establish a regional anesthesia service in the University Teaching Hospital of Kigali (CHUK). CHUK is the largest hospital in Rwanda (staffed by 3 anesthesiologists, 4 anesthesia residents, and 20 non-physician anesthesia providers, also called “technicians”). The implementation of the regional anesthesia service was conceived through collaboration between Canadian (MH, PL) and Rwandan (JN, JU) anesthesiologists in September 2015. A strategic plan was drafted to establish the service according to Paul Farmer's 4Ss—stuff, space, staff and systems [13]. Implementation began in January 2016. This involved:

*Staff —* MH and JN co-led the establishment project. All anesthesia staff participated in a 12-week regional anesthesia curriculum involving didactic, workshop, simulation and bedside teaching. A roster was created whereby one consultant or resident and one technician were assigned to the regional block room daily. Multidisciplinary education sessions were offered to perioperative surgical and nursing staff.

*Space —* A regional block space was created in the pre-operative waiting area. This room was equipped with a stretcher, block cart, a pulse oximeter, protocols and material for data recording and staff education.

*Stuff —* CHUK already had two ultrasound machines for use in theatre. A three-month supply of regional anesthesia equipment (e.g, block needles, local anaesthetic, ultrasound gel, and probe covers) was donated by Canadian anesthesiologists. During the implementation phase, Rwandan staff (JN, JU) advocated to hospital authorities to fund the procurement of regional anesthesia equipment for longer-term use.

*Systems —* patients booked for limb surgery were identified prior to arrival and directed to the block room for assessment. Anesthesia staff allocated to the block room were responsible for performance of the procedure, documentation, and follow up in the post-operative ward 24-hours later.

The official implementation period concluded after three months in April 2016. At this point, staff at CHUK had undergone the 12-week training period in regional anesthesia. Most patients undergoing emergency limb surgery received spinal and/or peripheral nerve blockade. All consultants and residents at CHUK were regularly performing blocks under supervision, while two residents attained competence to perform blocks without supervision, and to teach other residents.

**Interview participants:** following establishment, we used purposive sampling to obtain a variety of perspectives on regional anesthesia implementation [14]. Interview participants included anesthesia providers (anesthesiologists, residents, technicians) and other perioperative surgical staff (surgeons, perioperative nursing) who performed clinical and non-clinical (administrative, education) roles. By interviewing a range of participants, we aimed to capture divergent opinions for a nuanced understanding of the research question. Recruitment commenced at the end of the implementation phase and continued until at least three interviews were conducted without new substantive issues emerging. This defined our stopping criterion, at which point we concluded that data saturation had been reached [15].

**Figure 1:**
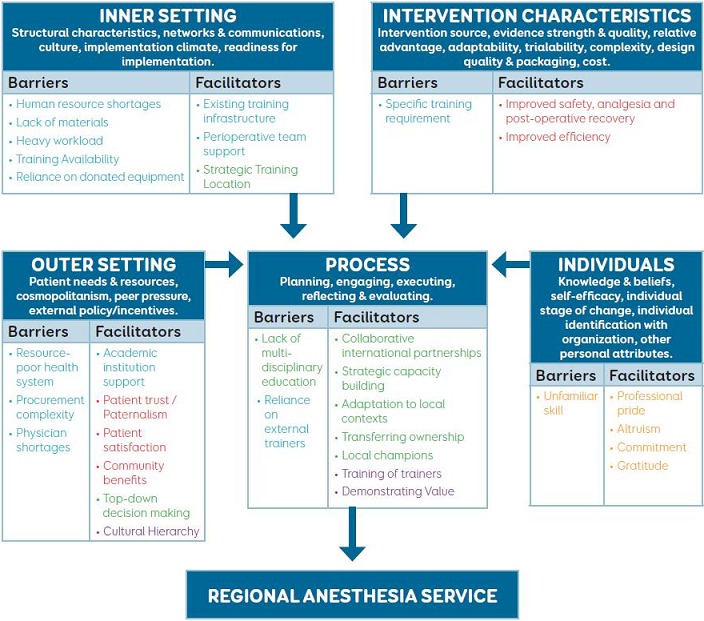
CFIR applied to the implementation and evaluation of a regional service in a CHUK

**Interviews:** following written consent, two study investigators of the interview team (MH, JN, FN; at least one of whom was a Rwandan) conducted interviews using a semi-structured interview guide, informed by the CFIR. Consolidating constructs from 19 frameworks and models in the implementation science literature [11], the CFIR comprises five domains: intervention characteristics, outer setting (i.e, the political, economic, and social context in which the organization operates), inner setting (i.e, the organization in which the implementation takes place), characteristics of the individuals involved, and the process of implementation (Figure 1). Single interviews were conducted in the workplace and lasted 30-45min. The semi-structured format has several advantages: it allows participants to respond freely to concepts; it provides enough structure that interviews can be completed in a timely manner; and it offers leeway for the interviewer to explore issues that arise but are not covered by the interview guide [16]. Interviews were conducted in English, French and Kinyarwanda, based on the comfort of the participant. All interviews were audio-recorded, translated (if necessary) and transcribed verbatim. All identifying information was removed and transcripts were not returned to participants.

**Data analysis:** transcripts were imported into NVivoTM (QSR International, Victoria, Australia) for coding and analysis. Two members of the research team (MH, RU) used three pilot interviews to independently and iteratively develop a coding scheme through both inductive and deductive methods. These codes were assigned a definition, inclusion/exclusion criteria, and appropriate quote examples. The agreed codes were categorized according to the five domains of the CFIR (Figure 1). This scheme was used to code the remaining transcripts. During the coding process, the coders met with an additional team member (PL) to discuss results and resolve divergent coding through the re-examination of transcripts. Through multiple team meetings, we analyzed code frequency, relationships between codes, and examined individual quotes to develop major thematic categories. These were reviewed and refined by the whole research team with reference to the original coding data, until agreement was reached that the themes accurately reflected the interview data. The research team represented anesthesiologists from Canada and Rwanda with a variety of roles – clinical, educational, and researchers – as well as a scientist with expertise in implementation science (RU). This brought a range of perspectives with which to interpret the interview data.

## Results

We interviewed 18 participants (Table 1), a number consistent with previous studies employing the CFIR [17-20]. All participants approached agreed to be interviewed. Themes that emerged from the interviews were: patient experience and outcomes; professional satisfaction; human and material resources; and local engagement for sustainability. Figure 1 delineates specific barriers and facilitators according to theme and relates these themes back to the CFIR.

**Table 1:** interview participant characteristics

Role	Number
Anesthesiologist	4
Anesthesia Technician	4
Anesthesia Resident	4
Surgeon	3
Surgical Resident	1
Nurse	2

**Patient experience and outcomes:** in a resource-limited context where equipment failure is frequent and essential medications are often unavailable, participants conveyed that Rwandan patients perceive anesthesia-related morbidity and mortality to be high. *“[Patients] are scared because many years ago when the patient was under general anesthesia, they often didn't wake up”* (Anesthesia Technician). They report that their patients are fearful of general anesthesia. *“The people are scared of general anesthesia because we don't have the anesthesiologist so people were not good in giving the anesthesia, and they (patients) lose their life”* (Nurse).

Yet, participants also commented that these same patients inherently trust physician decisions. Despite public awareness of regional anesthesia being low, patients readily consented for these techniques. *“In Rwanda people are very receptive. They trust their physician there is no way for them to refuse regional”* (Anesthesiologist). Participants considered that regional anesthesia improved patient safety and analgesia compared to general anesthesia. *“We have complications of missing airway control, and missing pain control in recovery. Patients are crying from pain. And we realized that regional can be a solution for that”* (Anesthesiologist). Participants explained that enhanced post-operative recovery and reduced staff workload improved perioperative efficiency. *“There are patients who actually don't need to be admitted and can benefit. I remember this helped me out with a patient I had seen and was failing to get a bed for him. We got a regional block, and we took the patient in the minor theatre room, and then we managed to do the procedure. He went home the same day and he was very happy”* (Surgeon).

Finally, Rwandan patients heavily rely on their families to provide transport, food and medical equipment for their hospital stay. Participants commented on benefit for the wider community from enhanced recovery and reduced length of stay: *“If someone is poor and coming for regional anesthesia, he will get home and work faster. I think this will help. If I am a mom and alone with my children, and can get home soon without hospital delay, this will have an influence on the whole family”* (Anesthesia Resident).

**Professional satisfaction:** participants conveyed pride in introducing innovative techniques in Rwanda. This contributed to positive morale among perioperative staff. *“When we started giving regional anesthesia, surgeons and other people were surprised it is amazing for them to see us giving anesthesia and see the patients being operated on while awake”* (Anesthesia Resident). Altruistic views were expressed in the goal to see regional anesthesia spread from CHUK to the rest of the country. In acknowledging the support of external volunteers into the regional service, participants conveyed a responsibility to work hard to bring this investment into fruition. *“CASIEF is doing also a great job through teaching residents and even the curriculum but we have also to do our job”* (Anesthesia Resident).

**Human and material resources:** despite the advantages of regional anesthesia, participants outlined major challenges related to the shortage of staff and materials. Anesthesia staff felt stretched by a demanding clinical workload. *“We are working in six ORs, two in gyne-obs, sedations for CT scans, patient visits, sedation for colonoscopy we have to be in the recovery room, in ophthalmology, we also have to be on call in other departments of CHUK for doing IV lines”* (Anesthesia technician). This resulted in two major challenges to establishing the regional anesthesia service. Firstly, it was almost impossible to routinely staff the regional block room with one supervising anesthesiologist, one resident, and one anesthesia technician. Secondly, staff had limited time available for learning regional anesthesia. By the end of our 3-month establishment phase, only one anesthesiologist, four residents and two technicians had attained independent proficiency in performing basic ultrasound-guided peripheral nerve blocks unsupervised. Anesthesiologists felt they had little time for training after covering their clinical commitments. *“When I am the only anesthesiologist in theatre, I have to cover all 6 cases, not just regional anesthesia so it is difficult to concentrate on regional”* (Anesthesiologist). The overwhelming strain of staff shortages was attenuated by the participants' commitment. *“I am sometimes about to put small babies to sleep. Then I say, “I'm coming,” so I [first] ran to the block room, reviewed the patient and did everything [the regional block]. After, I ran back to the baby, then ran back to see if the block was working. It is not easy, but you have to fight for it”* (Anesthesia Resident). Most participants suggested long-term capacity building to address these issues: encouraging residents with regional anesthesia experience to teach staff who lacked the time to train (a revolutionary concept in a hierarchical work culture); inviting staff from district hospitals to train at CHUK in order to reduce future referrals; establishing a resident rotation system at CHUK for regional anesthesia; and scheduling technicians to train and disseminate regional expertise. *“The vision is in two years those residents will be comfortable [in regional]. And they can also do workshops for all of the anesthesiologist staff in Rwanda”* (Anesthesia Resident)

The human resource shortages were compounded by a lack of equipment required for regional anesthesia. “We are a hospital that receives many patients who cannot pay and…the hospital doesn't have the materials they need like drugs. The establishment of this system is a difficult situation” (Anesthesia Resident). The shortages included but were not limited to: procedural equipment; equipment maintenance; drugs; patient monitors; and resuscitation equipment. During the establishment phase, CHUK was reliant on international donations. Participants identified the challenge of reliance on donations to local sustainability. “The problem is also spare parts. We can have the block needles today, but tomorrow we don't have them. We can have a good working ultrasound today, and tomorrow we don't have it. That is our big concern” (Anesthesia technician). Participant optimism was dampened by the complexity and rigidity of equipment procurement at CHUK. Barriers described included: approval requirements from multiple governing bodies; restriction of procurement responsibility to one government distributor with a minimum time lag of 6 months; and a lack of local suppliers of specific regional equipment. Participants emphasized that equipment shortages are potentially solvable through local advocacy, especially at a hospital management level. “You have authorities who have the job to find what is needed. And if they can find what is needed, it makes our job easier maybe they don't understand yet the importance of having a regional service that is sustainable. So we have maybe to advocate” (Anesthesiologist)

**Local engagement for sustainability:** while participants readily acknowledged the importance of collaborative international relationships for initiation of the regional anesthesia service, local staff emphasized the need to transfer the initiative and establishment process to local hands. *“So we can't succeed if local people are not well involved. It's like building with a false foundation. It will fall down surely”* (Anesthesia Resident). This was the basis for choosing an institution staffed by the country's academic and clinical leaders. As CHUK is the largest teaching hospital in the country, participants commented on its strategic location and function for the teaching and spread of regional anesthesia in Rwanda. *“As CHUK is a teaching hospital, we have anesthesia residents, and anesthesia technicians who are training here. So establishing regional anesthesia here will have a good impact on spreading regional anesthesia to other hospitals in different parts of our country'’* (Anesthesiologist). To promote Rwandan sustainability, department heads were involved in drafting a strategic plan for establishment of regional anesthesia. This meant setting realistic minimum standards for equipment and training. For example, contrary to most North American guidelines, anesthesia technicians, who provide most anesthesia services in Rwanda, were included in regional block training. This inclusive approach was considered important to dissemination of regional anesthesia practice. *“We don't have a lot of anesthesiologists so it should be a kind of task sharing with other anesthesia providers like anesthesia technicians. Select many of them to teach those skills so they can perform them safely and be able to sustain this service even if there is no anesthesiologist around”* (Surgeon).

Participants spoke of the need to nominate a local leader to train, advocate and inspire fellow staff in regional anesthesia for sustainability. *“[Anesthesiologist] has done the training outside the country and now he's here, he can start advocating that they have knowledge from outside, to save Rwandans. He is trained for this. He could be the center of advocacy”* (Anesthesia Resident). Participants emphasized the need to involve higher-tier academic organizations in promoting regional anesthesia training, such as UR and the Ministry of Health. *“The program director in anesthesiais involved and is accepting of having some residents do rotations in regional here in CHUK. I think different organizations in our country, especially the university, is committed to continue to help The Minister of Health can help us to implement the policy and education”* (Anesthesiologist). Participants conveyed the need to engage local stakeholders to foster sustainability of regional anesthesia, and suggested showing the value of regional anesthesia to a broad range of healthcare providers (e.g. operating room nurses, hospital ward personnel), administrators and procurement officers. *“We must have communication between the surgeon, the theatre managers not only for anesthesia, it's all the team We need the communication”* (Nurse).

Participants felt this advocacy was most effectively performed via a ‘top-down’ approach, given the strong hierarchical nature of the Rwandan health system. *“We need to involve the authorities from the hospital. Tell them what we are doing now and where we are and what we need to make the service sustainable. But otherwise, if we keep this service only for ourselves and we don't try to enroll the authorities, there is no way to make it sustainable”* (Anesthesiologist). Despite the limitations of the engagement process, participants recognized clear support and high morale amongst individuals and departments who had been engaged. *“When you see how you are as a team on a patient, training residents, training anesthesiologists with technicians, I am happy to see this kind of training it is done as a team and I appreciate it”* (Surgeon).

## Discussion

While implementation teams in high- and low-resourced settings have used the CFIR to understand and evaluate implementation processes [17-20], this study is unique in examining the adaptation of a well-established anesthetic technique into a low-resource setting. As our intervention was informed by the CFIR, the key findings from this study are grouped according to its 5 domains of implementation (Figure 1).

There is an increasing push for global health policy to be informed by evidence-based strategies [21]. In an evaluative pilot review of 15 studies on 3 specific global health interventions, Luoto *et al.* showed that the reporting of implementation processes and context descriptors was mostly fair to poor [21]. These factors make direct comparison with other studies difficult. Nevertheless, the CFIR constructs identify key principles applicable to this study [11]: externally-initiated interventions are more difficult to sustain than internal (‘grass-roots’) efforts [22]; low-cost locally funded interventions predict success over those that are costly and externally supported [23]; alignment of the intervention with the country's overall health goals is essential [24]; and assessing locally-focused data on barriers, facilitators and patient needs is required prior to establishment [22]. English *et al.* emphasized similar strategic recommendations when implementing a theory-informed pediatric service in Kenya. This included: engaging multiple stakeholders; gaining government approval; and empowering credible local partners [25]. An important distinction in low-resource countries such as Rwanda is the need and ability to engage support at the highest level (e.g. the Ministry of Health), unlike similar interventions in high-resource contexts, which will aim at a much lower level (e.g. institutional or departmental).

Despite these recommendations, there are several issues worthy of deeper discussion. In contrast to CFIR recommendations [11], participants acknowledged that meaningful establishment of the service would have been difficult without external assistance. Unlike well-resourced contexts, resource-poor health systems are unable to provide the initial investment required for structured service implementation and rely on external donations. Local staff may also lack the knowledge to effectively advocate for local ‘buy-in’ of such implementation projects. Thus, initial donations may help demonstrate the intervention value and increase likelihood of future local investment. Indeed, participants expressed gratitude for this assistance and acknowledged an obligation to transfer responsibility to local ownership to sustain the regional anesthesia service.

This same sense of gratitude may have biased our results, as our data spoke of regional anesthesia-specific barriers in relative paucity. For example, very few of the participants mentioned common misgivings expressed in Western countries such as: time taken for block learning; slowing of list turnover; consequences of failed blocks; potential complications such as local anesthesia toxicity and nerve damage; and patient anxiety [26]. Both local and international authors of this study observed difficulty amongst Rwandan staff in expressing negative opinions about volunteer intervention for two reasons: a sense of gratitude, and the non-confrontational nature of Rwandan verbal communication. Future studies could mitigate this by limiting interview data collection to local investigators. Likewise, while Rwandan patients' unquestioning consent to treatment was a facilitator for establishment of regional anesthesia, the authors acknowledge that medical paternalism and patient disengagement with clinical decisions is problematic.

Our study exemplifies the importance of transferring responsibility to local ownership. The relationship between CASIEF and UR fosters a trusting environment for collaborative planning between external volunteers and local staff. Pre-implementation planning visits by external staff were necessary to assess the human and material resource barriers in the inner and outer settings; to raise awareness and galvanize staff motivation; and to commence official planning with local leaders for establishment. For example, having acknowledged the difficulties in procuring regional equipment locally, we suggest gaining early support from hospital procurement officers through education and advocacy. Local sustainability planning should include agreement on a set timeframe of external resource provision. Overseas volunteers need to perform graded handover of responsibility, skills and resources to local implementation team members throughout the establishment phase. Formal local leadership, duty and role allocations must conclude this transfer process prior to the external volunteers leaving. Finally, follow-up mentorship support is required by external volunteers through long-distance communications and future visits.

The Rwandan ‘local champion’ exemplified the benefits of understanding knowledge translation in global health. One year prior to implementation, he was awarded a World Federation of Societies of Anaesthesiologists (WFSA) scholarship to train overseas in regional anesthesia for 6 months. Despite returning as the sole Rwandan skilled in regional anesthesia, he seldom performed or taught blocks, due to the numerous barriers to practice change. Within six months of implementation, this anesthesiologist regularly performed regional blocks for trauma at CHUK, taught regional anesthesia at UR, and organized a procurement plan for regional anesthesia equipment for CHUK.

Limitations of this study are that data were acquired only through interviews of health professionals and did not include patients receiving regional anesthesia. Our inductive approach of analysis did not cover all the 39 CFIR constructs. Other evaluative studies in global health contexts have effectively used mixed methods (interviews, field notes, quantitative outcome data) [18, 27]. This study team is concurrently performing a quantitative retrospective outcome study, which may be useful for future mixed method evaluation. As data were collected at the conclusion of the 3-month establishment phase, our study was unable to evaluate the major goal of sustainability. Finally, this study was conducted in a single low-resource institution and may not be representative of the implementation issues faced in other low-HDI countries.

## Conclusion

Our study identified barriers and facilitators to establishing a perioperative service in a low-resource setting and analyzed them using an established implementation framework. The CFIR demonstrates the dynamic interplay between the 5 domains of implementation, from which we have provided a broad framework to guide future establishment endeavors in low-resource contexts, including our attempts to improve this service in Rwanda. In this unique setting, we encourage future studies to build on and refine our findings.

### What is known about this topic

- Despite advantages over general anesthesia in terms of analgesia, recovery and cost, regional anesthesia is underutilized in low-resource settings due to a lack of infrastructure, trained staff and appropriate processing systems;
- Health policy should be informed by evidence-based strategies such as the CFIR, which emphasize: a pre-intervention needs assessment; alignment of the intervention with a country's overall health goals; and a locally-based funding model.

### What this study adds

- Global health interventions require focused support from high-level health organizations, more than the low-level institutional support that is emphasized in high-resource settings;
- Unlike interventions in well-resourced settings, external assistance is usually necessary for the establishment of new health services in low-resource settings;
- Unlike interventions in well-resourced settings, external assistance is usually necessary for the establishment of new health services in low-resource settings;

## Competing interests

The authors declare no competing interests.
